# A red-emitting carborhodamine for monitoring and measuring membrane potential

**DOI:** 10.1073/pnas.2315264121

**Published:** 2024-03-29

**Authors:** Anneliese M. M. Gest, Julia R. Lazzari-Dean, Gloria Ortiz, Susanna K. Yaeger-Weiss, Steven C. Boggess, Evan W. Miller

**Affiliations:** ^a^Department of Chemistry, University of California, Berkeley, CA 94720; ^b^Department of Molecular & Cell Biology, University of California, Berkeley, CA 94720; ^c^Helen Wills Neuroscience Institute, University of California, Berkeley, CA 94720

**Keywords:** physiology, fluorescence, imaging, voltage

## Abstract

Biological membrane potentials, or voltages, are a central facet of cellular life. Optical methods to visualize cellular membrane voltages with fluorescent indicators are an attractive complement to traditional electrode-based approaches, since imaging methods can be high throughput, less invasive, and provide more spatial resolution than electrodes. Recently developed fluorescent indicators for voltage largely report changes in membrane voltage by monitoring voltage-dependent fluctuations in fluorescence intensity. However, it would be useful to be able to not only monitor changes but also measure values of membrane potentials. This study discloses a fluorescent indicator which can address both. We describe the synthesis of a sulfonated tetramethyl carborhodamine fluorophore. When this carborhodamine is conjugated with an electron-rich, methoxy (-OMe) containing phenylenevinylene molecular wire, the resulting molecule, CRhOMe, is a voltage-sensitive fluorophore with red/far-red fluorescence. Using CRhOMe, changes in cellular membrane potential can be read out using fluorescence intensity or lifetime. In fluorescence intensity mode, CRhOMe tracks fast-spiking neuronal action potentials (APs) with greater signal-to-noise than state-of-the-art BeRST 1 (another voltage-sensitive fluorophore). CRhOMe can also measure values of membrane potential. The fluorescence lifetime of CRhOMe follows a single exponential decay, substantially improving the quantification of membrane potential values using fluorescence lifetime imaging microscopy (FLIM). The combination of red-shifted excitation and emission, mono-exponential decay, and high voltage sensitivity enable fast FLIM recording of APs in cardiomyocytes. The ability to both monitor and measure membrane potentials with red light using CRhOMe makes it an important approach for studying biological voltages.

## Significance

Biological membrane potentials are maintained by all forms of life. In electrically excitable cells, fast changes in membrane potential drive downstream events: neurotransmitter release, contraction, or insulin secretion. The ability to monitor changes in and measure values of cellular membrane potentials is central to a mechanistic understanding of cellular physiology and disease. Traditional modes for measuring membrane potential use electrodes, which are invasive, destructive, low throughput, and ill-suited to interrogate spatial dynamics of membrane potentials. Optical methods to visualize potentials with fluorescent dyes offer a powerful complement to traditional electrode approaches. In this study, we show that a red to far-red fluorophore can both monitor changes in and measure values of membrane potential in living systems.

Membrane potential plays central roles in the physiology of all living systems. Rapid changes in membrane potential initiate neurotransmitter release in neurons (1), prompt muscle contraction in cardiomyocytes (2, 3), and evoke insulin secretion in pancreatic β cells (4). In a related fashion, the value of the membrane potential—set largely by the difference in internal and external potassium ion concentration and the selective permeability of the plasma membrane to K^+^ ions—plays an outsized role in the physiology of these cell types, controlling their excitability by setting the distance to action threshold. Further, values of membrane potentials in non-electrically excitable cells may play important roles in signaling, differentiation, and cell cycle progression (5–7). Reflecting the importance of membrane potential, some 10 to 50% of oxygen consumption is directed toward setting the “resting” membrane potential through the action of the ATP-driven Na-K exchanger (8).

The primary mode of monitoring changes in and measuring values of membrane potential is patch-clamp electrophysiology. Despite the centrality of membrane potential to cellular physiology across all organ systems, electrophysiology is nonetheless invasive, low throughput, and difficult to implement across spatial scales. Electrophysiological analysis of multiple cells requires highly specialized instrumentation (9, 10), and interrogation of subcellular or intracellular membranes remain largely inaccessible except in limited cases (11–13).

To complement traditional electrode-based methods, we have been pursuing voltage-sensitive fluorophores (VoltageFluors, or VF dyes) that sense voltage via a photoinduced electron transfer (PeT)-based mechanism (14, 15). This approach can be adapted to a wide range of chemically synthesized fluorophores (16). Dyes with excitation and emission in the red region of the spectrum are especially useful, since they display lower levels of phototoxicity and minimize autofluorescence by avoiding excitation of endogenous chromophores; (17) have higher photostability; (17, 18) are compatible with optical indicators and actuators that use blue or green light; are well matched to commercially available powerful LEDs; and pair well with the wavelength-dependent quantum efficiency of typical sCMOS detectors, which falls off dramatically beyond 650 nm (19, 20). These characteristics make VF dyes well-suited for tracking fast membrane potential changes associated with action potentials (APs) in neurons (AP duration ~2 ms) or cardiomyocytes (AP duration ~100 ms).

We previously showed that VF dyes can be used to estimate values of membrane potential (21). Using fluorescence lifetime imaging microscopy (FLIM) to measure the fluorescence lifetime of VF dyes in cell membranes, we can estimate millivolt values of membrane potential, since fluorescence lifetime is more resistant than fluorescence intensity to variations in dye loading, fluctuations in excitation power, or bleaching (22). Our initial efforts with fluorescein-based VF2.1.Cl showed that FLIM enables membrane potential value estimation with 19× or 8× improved resolution compared to existing FLIM or ratio-based methods (21). However, the long acquisition times required for FLIM, coupled with the blue-light excitation of VF2.1.Cl means that even low levels of phototoxicity become limiting in sensitive systems (like cardiomyocytes or neurons) (23). Further, VF2.1.Cl has a two-component fluorescence lifetime decay, complicating analysis and requiring longer photon collection times to achieve accurate fitting, at the cost of speed and possible phototoxicity (24, 25).

Here, we describe a VF dye based on a tetramethyl carborhodamine (TMCRh) (26) scaffold. The carborhodamine indicators have red excitation and emission profiles and show high voltage sensitivity. One indicator in particular, a Carbo Rhodamine with a methoxy (OMe) wire, or CRhOMe (“chrome”), can detect rapid changes of membrane potential in hippocampal neurons with improved signal-to-noise ratio (SNR) compared to BeRST 1, our previous best in class. CRhOMe also possesses a long, monoexponential fluorescence lifetime, which is both simpler to fit and requires fewer photons for an accurate fit, dramatically improving its performance for tracking membrane potential values and changes using FLIM and allowing faster imaging. The unique photophysical properties of CRhOMe—high voltage sensitivity, long, monoexponential fluorescence lifetime (~3 ns), emission above 640 nm, and excellent photostability—substantially improve its performance compared to VF2.1.Cl in FLIM, making CRhOMe an exceptionally promising candidate for measuring membrane potential values and changes with FLIM.

## Synthesis

**Scheme 1.**
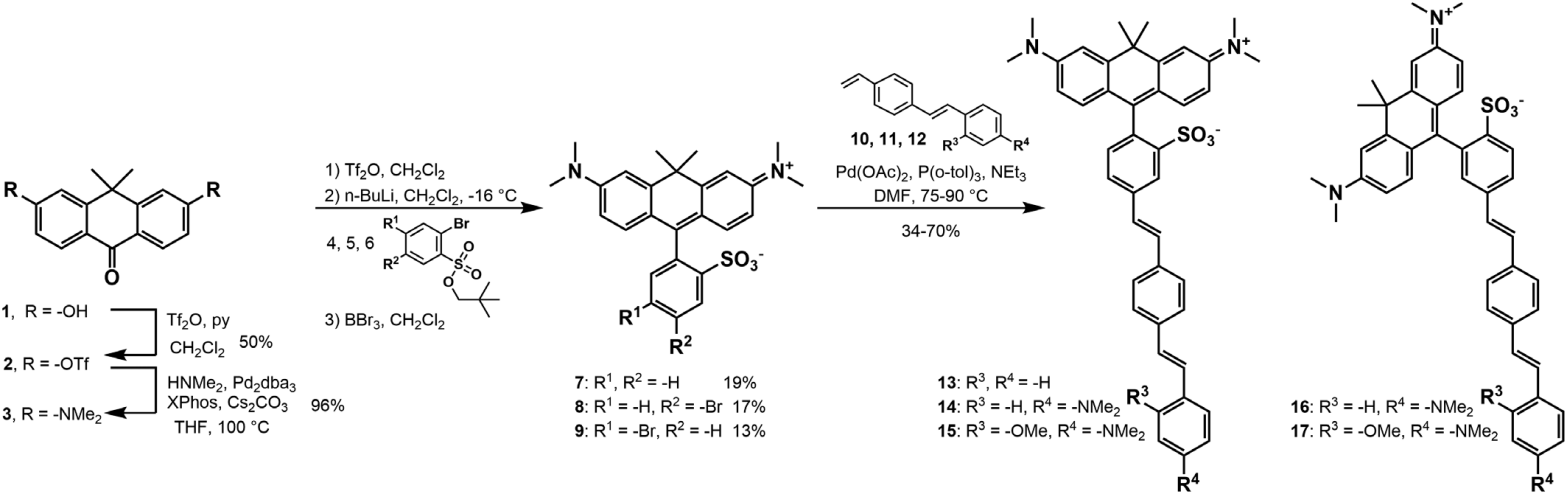
Synthesis of carborhodamine fluorophores (**7**–**9**) and voltage dyes (**13**–**17**).

We synthesized three sulfonated carborhodamine fluorophores (**7**–**9**, Scheme 1) and five carborhodamine voltage indicators (**13**–**17**, Scheme 1). The synthesis of sulfonated carborhodamine fluorophores begins with the triflation of anthracenone **1** (26) with trifluoromethanesulfonic anhydride and pyridine in dichloromethane to give **2** in 50% yield (Scheme 1). Triflate **2** is then subjected to a Buchwald–Hartwig reaction with dimethylamine to yield **3** in 96% yield. This cross-coupling route was previously established to install amino groups on fluorescein derivatives to access rhodamines (18, 26–28). To install the *meso* aromatic ring bearing a sulfonate group, we followed conditions used in the synthesis of BeRST 1 (18). Compound **3** is treated with triflic anhydride, then reacted with the organolithiums derived from **4**, **5**, or **6** with *n*-butyllithium in dichloromethane at −16 °C to yield sulfonated carborhodamines **7**–**9** in 13 to 17% yields (Scheme 1). Sulfonated carborhodamines **8** and **9** are then subjected to a Heck reaction with styrenes **10**, **11**, or **12** to yield TMCRh voltage indicators **14**–**17** and **13** (R^3^ = R^4^ = H), which lacks the alkyl-substituted aniline required for voltage sensitivity (29).

## Spectroscopic Characterization

We examined the photophysical properties of the TMCRh fluorophore **7** and dyes **13**–**17**. Sulfonated TMCRh **7** displays a λ_max_ of 610 nm in both methanol (ε = 129,000 M^−1^ cm^−1^) and EtOH with 0.1% TFA (ε = 125,000 M^−1^ cm^−1^) and an 11-nm shift in aqueous buffer with a λ_max_ of 621 nm (ε = 129,000 M^−1^ cm^−1^, PBS (Phosphate Buffered Saline), pH 7.4, with 0.1% sodium dodecyl sulfate (SDS), above the critical micelle concentration for phosphate buffered solutions) (30) (*SI Appendix*, Fig. S1). In alcoholic solvents, the λ_em_ is 633 nm, and in aqueous buffer, the λ_em_ is 641 nm. Sulfonation of TMCRh shifts absorbance and emission to the blue by ~16 nm compared to carboxylated tetramethylcarborhodamine (28). We observed a similar bathochromic shift for the comparison between sulfonated carbofluoresceins (31) and carboxy-substituted carbofluoresceins (26).

**Fig. 1.**
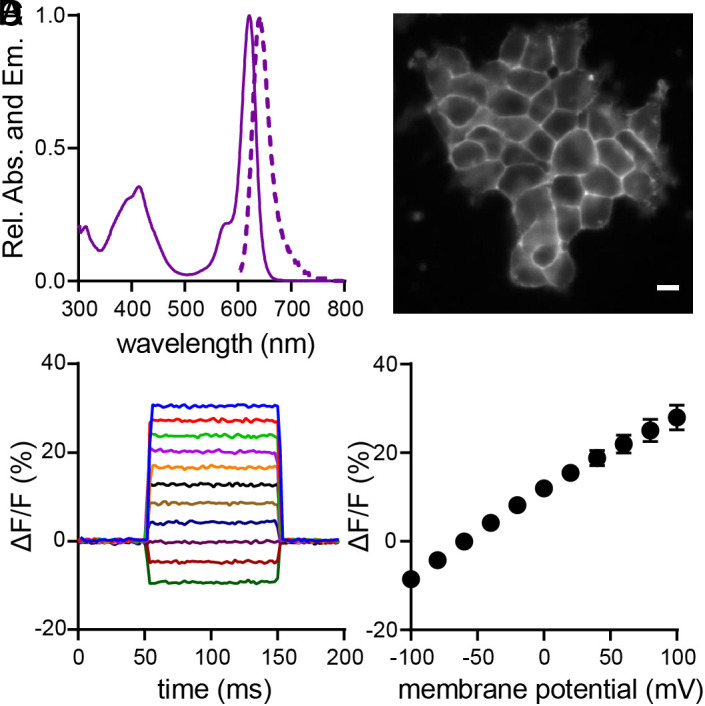
Characterization of CRhOMe (**15**) voltage sensitivity in HEK293T. (*A*) Absorption (solid line) and emission (dashed line) spectra of CRhOMe (**15**) in PBS, pH 7.4, 0.1% SDS. (*B*) Epifluorescence image of a group of HEK293T cells stained with 500 nM CRhOMe. (Scale bar is 10 µm.) (*C*) Change in fluorescence for a single HEK293T cell under whole-cell voltage clamp conditions in which the membrane potential was stepped from +100 to −100 mV in 20 mV increments. (*D*) Average fluorescence intensity change (%ΔF/F) observed across multiple voltage-clamped HEK293T cells. Error bars are ± SD for n = 8 cells.

The TMCRh voltage indicator derivatives **13**–**17** display similar spectral profiles in an aqueous buffer with a λ_max_ of 622 nm and a λ_em_ of 641 nm (Table 1, Fig. 1, and *SI Appendix*, Fig. S1). Fluorescence quantum yields (Φ) for putative voltage reporters **14**–**17** are low (0.038 to 0.096), while the voltage insensitive TMCRhZero (13) is much higher (Φ = 0.62), comparable to the TMCRh fluorophore **7** alone (Φ = 0.71). The 6- to 18-fold decrease in Φ for **14**–**17** compared to **13** indicates a high degree of PeT from the aniline donor to the fluorophore.

**Table 1.** Photophysical properties of carborhodamine dyes

Compound	R^3^	R^4^	λ_max_/nm^*^	λ_em_/nm^*^	Φ^a/b^	ΔF/F/100 mV^‡^	Relative brightness^§^
TMCRh-sulfonate **7**	n/a	n/a	621	641	0.71/0.75	n/a	n/a
TMCRhZero **13**	H	H	622	641	0.62/0.61	0.3% ± 0.1%	3.9 ± 0.12
TMCRh.H **14**	H	NMe_2_	622	642	0.096/0.087	1.2% ± 0.4%	1.0 ± 0.04
CRhOMe **15**	OMe	NMe_2_	622	642	0.038/0.027	18% ± 2%	3.8 ± 0.13
isoTMCRh.H **16**	H	NMe_2_	621	641	0.050/0.057	1.5% ± 0.2%	5.4 ± 0.22
isoCRhOMe **17**	OMe	NMe_2_	621	641	0.058/0.054	9% ± 2%	17 ± 0.84

^*^Determined in PBS, pH 7.4, 0.1% SDS.

^†^Determined in MeOH.

^‡^Voltage-clamped HEK cells. Error is ± SD for n = 4 to 7 cells.

^§^Determined in HEK cells. Error is ± SEM for n = 4 coverslips (>100 cells per coverslip) for relative brightness.

## Voltage Sensitivity in HEK cells

All aniline-containing voltage reporter derivatives (14–17) stain cellular membranes and show V_mem_ sensitivity in HEK293T cells (Fig. 1 *C* and *D* and *SI Appendix*, Figs. S1 and S2). TMCRh voltage indicators with the unsubstituted wire (**14**, **16**, R^3^ = H) display lower voltage sensitivity than their corresponding OMe-substituted wire counterparts (**15**, **17**, R^3^ = OMe). Compound **15** has a voltage sensitivity of 18% ± 2% ΔF/F per 100 mV and its isomer **17** has a voltage sensitivity of 9% ± 2% ΔF/F per 100 mV. We observe the highest voltage sensitivity in **15** and no voltage sensitivity in TMCRhZero **13** (0.3 ± 0.1%). The improved voltage sensitivity of the OMe-substituted indicators (15, 17) compared to unsubstituted indicators (14, 16) is similar to the trends for rhodamine-based voltage reporters (RhoVRs) (32, 33). One difference between RhoVRs and the current indicators is that, for RhoVRs, the dyes with a *meta* configuration between the fluorophore and wire are the most sensitive; (32, 33) whereas this is not the case for the current carborhodamine indicators. Because of its superior ΔF/F, we selected compound **15**, a Carbo Rhodamine with a OMe wire, or CRhOMe (chrome), for further investigation in subsequent experiments, including fluorescence lifetime imaging studies, along with TMCRhZero as a voltage-insensitive control compound.

## Performance in Neurons

**Fig. 2.**
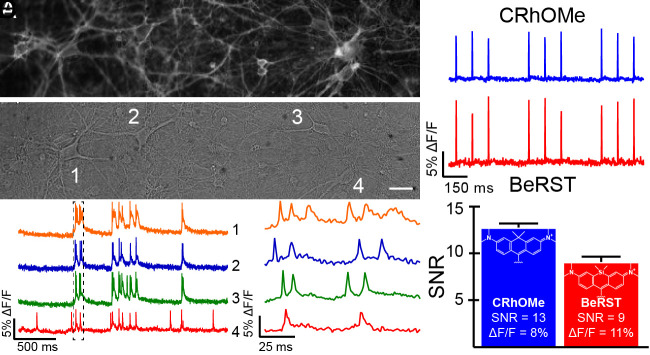
CRhOMe tracks spontaneous and evoked APs in cultured neurons. (*A*) Wide-field microscopy fluorescence and (*B*) DIC images of cultured rat hippocampal neurons stained with CRhOMe (500 nm, 30 min). (Scale bar is 20 μm.) (*C*) Optical traces of spontaneous activity of the neurons in panels (*A* and *B*) recorded at 500 Hz and shown as ΔF/F vs. time. (*D*) Highlighted APs from panel (*C*). (*E*) Plots of ΔF/F vs. time for neurons stained with either CRhOMe (blue) or BeRST 1 (red) and then subjected to field stimulation to evoke AP responses. (*F*) Comparison of SNR for CRhOMe (blue) and BeRST 1 (red) in neurons stimulated as in panel *E*. Data represent mean ± SEM for n = 50 or 21 neurons for CRhOMe and BeRST 1, respectively. Structures of the fluorophores for CRhOMe and BeRST 1 are provided for comparison purposes.

CRhOMe **15** stains cell membranes of dissociated rat hippocampal neurons and reports spontaneous APs from multiple cells (Fig. 2). Field stimulation of neurons labeled with CRhOMe reveals that CRhOMe responds to evoked APs with a voltage sensitivity of 8.2% ± 1.7 (1.85 W/cm^−2^; SNR = 22 ± 2, *n* = 22 cells, *SI Appendix*, Fig. S3). When comparing **15** to BeRST 1 using minimal light power (0.93 W/cm^−2^), CRhOMe reported evoked APs with 8.4% ± 0.3% and SNR = 13 ± 0.4 (*n* = 50 cells), comparable or better than BeRST 1, which responded to evoked APs with 11% ± 0.7% and SNR = 9 ± 0.5 (*n* = 21 cells, Fig. 2 and *SI Appendix*, Fig. S4). At all light powers examined, CRhOMe has significantly higher SNR than BeRST 1 (*P* = 0.024, paired *t* test) and a trend toward a lower nominal ΔF/F (9% vs. 11%, *P* = 0.068, paired *t* test, Fig. 2 and *SI Appendix*, Fig. S4). CRhOMe shows excellent resistance to photobleaching, comparable to BeRST 1, the existing best-in-class (*SI Appendix*, Fig. S4) (34). At higher light powers, around 13 W/cm^2^, or approximately 10× higher than that used for routine neuronal imaging in our lab (35), both BeRST 1 and CRhOMe display the previously reported phototoxicity associated with extended illumination (*SI Appendix*, Fig. S4 *G* and *H*).

## Lifetime Studies in Cells

**Fig. 3.**
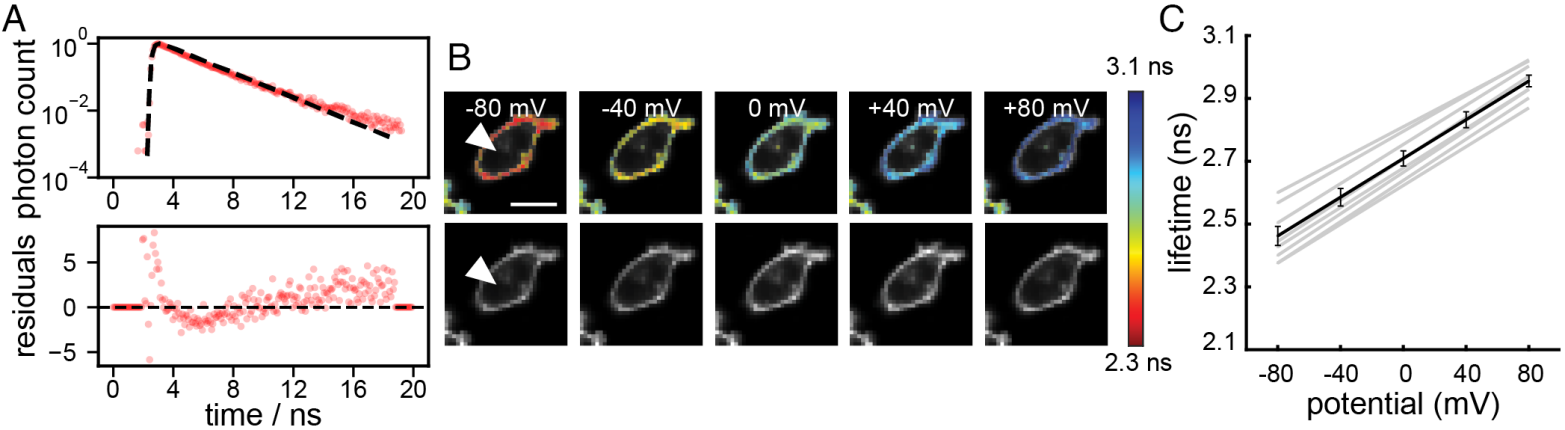
CRhOMe displays single-exponential fluorescence lifetime decays and a linear fluorescence lifetime-voltage relationship. (*A*) Plots of fluorescence lifetime decay expressed as normalized photon counts (log scale) vs. time for CRhOMe. Circles indicate photon count data; dashed line is a single exponential decay. Weighted residuals are plotted in the lower graph. (*B*) Lifetime (colored) and intensity (grayscale) images of HEK293T cells voltage-clamped at the indicated potentials. The lifetime heatmap is scaled from 2.3 to 3.1 ns. (Scalebar is 20 µm.) Arrowhead indicates the voltage-clamped cell. (*C*) Plot of CRhOMe fluorescence lifetime vs. membrane potential in HEK293T cells. Gray lines are individual cell calibrations (n = 17). The average calibration is in black. Error bars are mean ± SEM.

We recorded the time-resolved fluorescence decay of the TMCRh fluorophore **7** (1 µM in water) with time-correlated single photon counting (TCSPC) FLIM on a point scanning confocal microscope. The time-resolved fluorescence decay was well described by a single exponential model and exhibited a lifetime of 3.06 ± 0.02 ns (mean ± SEM of 16 measurements, *SI Appendix*, Fig. S5). We then measured the fluorescence lifetime of CRhOMe in living cells (Fig. 3*A*), which was also well described by a single exponential decay. We observe a mean lifetime of 2.57 ± 0.01 ns in HEK293T at rest (mean ± SEM of 132 cell groups) and a mean lifetime of 2.60 ± 0.02 ns in serum-starved A431 cells (mean ± SEM of 18 cell groups). For the voltage-insensitive control compound TMCRhZero in HEK293T (*SI Appendix*, Fig. S7), we again observe a single exponential decay with a lifetime of 3.70 ± 0.01 (mean ± SEM of 103 cell groups). Lifetime data collected with different instruments or at different excitation wavelengths give the same resting lifetime potentials in HEK293T cells (*SI Appendix*, Fig. S6).

As a comparison, we also investigated the fluorescence lifetime in HEK293T cells of our existing best-in-class far-red VF, BeRST 1. Unlike CRhOMe, BeRST 1 is not well described by a single exponential decay (*SI Appendix*, Fig. S5*B*) and was instead fit with a two-component exponential decay. We report its lifetime as the amplitude-weighted average of the two lifetime components, as we have done for previous green VoltageFluors in FLIM (21). In HEK293T cells, BeRST 1 exhibits a weighted average lifetime of 1.80 ± 0.01 ns (mean ± SEM of 56 cell groups). This one-component decay for CRhOMe is a critical advantage in FLIM studies. Simpler, one-component decay models can be confidently fit with at least an order of magnitude fewer photons than more complicated multi-component models (36, 37). Error analysis on simulated, ideal fluorophores shows that for a monoexponential decay, a relative error of 6% can be achieved with as few as 400 photons—under the same conditions, a bi-exponential decay requires 10,000 to 20,000 photons to achieve the same accuracy (37). Because of its monoexponential decay, CRhOMe FLIM data can therefore be acquired faster and with lower phototoxicity than either BeRST 1 or our previously studied green VoltageFluors, due to the lower number of photons required to reliably perform lifetime fitting (36, 37).

We additionally measured lifetimes across a range of dye loading concentrations for both CRhOMe and BeRST 1 to look for a concentration-dependent decrease in τ_fl_ (*SI Appendix*, Fig. S7), which we previously observed with green VoltageFluors (21, 29). All subsequent experiments were conducted at concentrations below the point where concentration quenching was observed (approximately 500 nM in HEK293T culture, with most experiments done at 300 nM dye).

## Calibration of CRhOMe in Cells with Electrophysiology and Gramicidin Treatment

We generated a voltage-τ_fl_ calibration using whole-cell, patch-clamp electrophysiology, and simultaneous FLIM imaging to determine the voltage dependence of CRhOMe fluorescence lifetime. HEK293T cells were treated with 300 nM CRhOMe and then voltage-clamped at −80, −40, 0, +40, and +80 mV, and the fluorescence lifetime was recorded. Individual measurements demonstrate a linear response of lifetime to changes in voltage (Fig. 3 *B* and *C*). Consistent responses among individual measurements allow for the development of an average calibration to represent the overall expected change in lifetime (in ps) per change in voltage (in mV). In HEK293T cells, CRhOMe exhibits a sensitivity (slope of the calibration) of 3.09 ± 0.09 ps/mV, with a lifetime at 0 mV (the y-intercept of the calibration) of 2.71 ± 0.03 ns. Applying this calibration to our measured lifetime of HEK293T cells at rest, the mean resting membrane potential across all measurements is −41.0 ± 3.5 mV (*SI Appendix*, Fig. S6*C*, mean ± SEM), consistent with previous FLIM-based optical estimates of HEK293T resting potential values determined with VF2.1.Cl (−47 ± 5 mV) (21) or electrophysiological measurements (38–40), which range from −52 mV (39) to −35 mV (40) [average is −44 mV (21)]. The voltage-insensitive control compound, TMCRhZero, does not exhibit a change in lifetime to an applied voltage (*SI Appendix*, Fig. S8).

To provide a secondary, non-electrophysiological calibration for systems where electrophysiology may be impractical, we turned to the Na^+^/K^+^ ionophore gramicidin (41) (*SI Appendix*, Figs. S9 and S10). Because it increases the permeability to both Na^+^ and K^+^, we expect that gramicidin will depolarize cellular V_mem_ to approximately 0 mV. In HEK293T cells treated with 500 ng/mL gramicidin, the fluorescence lifetime of CRhOMe rose to 2.74 ± 0.09 ns (mean ± SD of 111 cell groups), which matches the 0 mV τ_fl_ of 2.71 ns determined by patch clamp calibration. We observed a similar increase in fluorescence lifetime in serum-starved A431 cells treated with gramicidin (*SI Appendix*, Fig. S9), whereas we observe minimal change in the fluorescence lifetime of TMCRhZero in gramicidin-treated HEK293T (*SI Appendix*, Fig. S10). Serum-starved A431 cells showed an increase in lifetime of about 215 ps from 2.61 ± 0.09 ns (mean ± SD of 27 cell groups) to 2.83 ± 0.06 ns (mean ± SD of 26 cell groups). Assuming that gramicidin treatment brings these cells to a V_m_ of zero and that the V_m_ of the cells is near the reported mean (−64 mV for A431 cells) (42), this suggests a sensitivity of approximately 3.5 ps/mV. This value is similar to the sensitivity determined by electrophysiology in HEK293T cells, with the slight disagreement likely due to either cell-type dependence of the calibration or errors in our assumptions about A431 V_m_ before and after gramicidin treatment. These data suggest that gramicidin may be appropriate for creating a reference point for calibrations in cell lines where a ground-truth patch clamping experiment is not feasible, although it requires assumptions about the resting V_m_ of cells and ability of gramicidin to fully zero the value of V_m_.

The magnitude of CRhOMe lifetime to changes in V_mem_ is comparable to that of VF2.1.Cl (previously reported sensitivity 3.5 ps/mV, 0 mV lifetime 1.77 ns in HEK293T) (21). The sensitivity of VF2.1.Cl in lifetime is the highest reported for a VF to date, despite the presence of probes with higher fractional voltage sensitivity (ΔF/F) (29). CRhOMe’s high sensitivity, combined with its attractive spectral properties and single component decay, indicated that CRhOMe would be a superior choice for FLIM quantification than the previously reported VF2.1.Cl or BeRST 1. We therefore investigated applications of CRhOMe lifetime as a reporter of absolute V_m_.

## Monitoring EGF (Epidermal Growth Factor)-Induced Hyperpolarization in A431 Cells

A key advantage of red-shifted voltage sensors is the ability to record for long periods of time with low phototoxicity. To evaluate CRhOMe FLIM in this context, we recorded the response of serum-starved A431 cells to re-introduction of EGF (500 ng/mL) for 15 min. We previously performed this experiment with the green VoltageFluor VF2.1.Cl (21). Using VF2.1.Cl, we were able to make six, intermittent V_mem_ measurements within a 15-min time window, with each measurement lasting 30 s. These previous experiments revealed a hyperpolarizing response to EGF stimulation, facilitated by the kinase activity of EGFR, dependent on internal Ca^2+^ release, and mediated by the action of the Ca^2+^-activated K^+^ channel, K_Ca3.1_ (21).

**Fig. 4.**
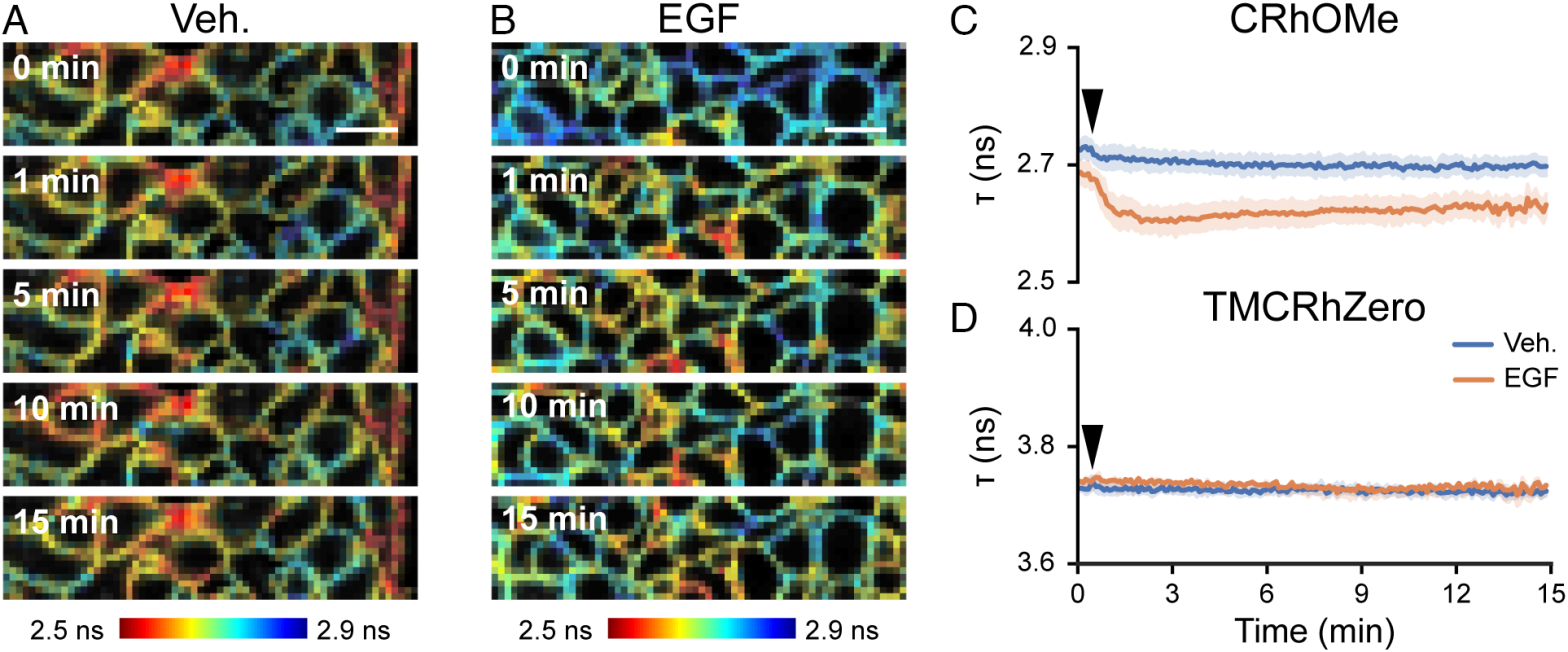
CRhOMe reports on EGF-induced hyperpolarization events in A431 cells with enhanced temporal and spatial resolution. Snapshots from a τ time series of serum-starved A431 cells treated with either (*A*) vehicle (imaging buffer) or (*B*) EGF. (Scale bar is 20 μm.) Each snapshot is acquired at the indicated time after starting the experiment. Vehicle or EGF is added 20 s into the experiment. (*C*) Mean lifetime of CRhOMe or (*D*) TMCRhZero across the full recording. Shading represents SEM. Vehicle or 500 ng/mL EGF was added at the black arrow. Sample sizes: CRhOMe Veh 5, EGF 5; TMCRhZero Veh 3, EGF 3.

Using CRhOMe as a voltage indicator, we were able to record continuously for 15 min, with each measurement lasting 5 s across a field of view 1.5 fold larger than the VF2.1.Cl study (Fig. 4 and *SI Appendix*, Fig. S11) for a total of ~180 V_mem_ measurements, compared to 6 measurements with VF2.1.Cl, an order of magnitude increase in total imaging time. In EGF-treated samples, we observe an immediate lifetime decrease of 75 ps on average (approximately a 21 mV change based on the gramicidin calibration discussed above) with a steady return to baseline that did not complete within 15 min (Fig. 4), consistent with the expected hyperpolarization (21). Samples treated with imaging buffer vehicle show no change in τ_fl_ of CRhOMe (Fig. 4*A*). The control TMCRhZero displayed a steady, slight shortening of the lifetime (Fig. 4*D*), which was identical when treated with either vehicle or EGF. The spatial distribution of lifetime values in both vehicle and EGF-treated cells is intriguing and hints at the possibility of heterogenous V_mem_ distributions in otherwise “uniform” populations of cells that have been reported elsewhere (43).

## FLIM to Monitor Cardiac APs

**Fig. 5.**
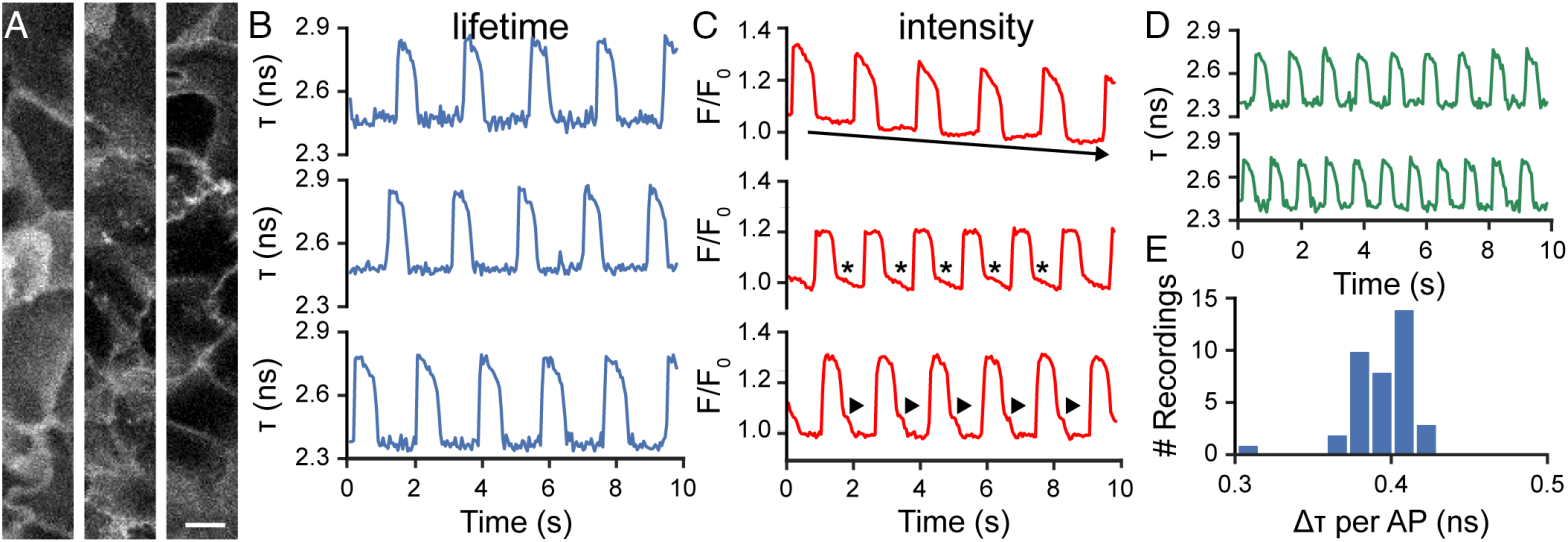
Fluorescence lifetime imaging of spontaneously beating cardiomyocytes using CRhOMe avoids artifacts associated with intensity-based imaging. (*A*) Representative confocal images of regions of iCMs used for 20 Hz lifetime imaging. Images here are fluorescence intensity only and were acquired for 400 ms (line averaged) to improve contrast. iCMs were stained with 500 nM CRhOMe. (Scale bar is 10 µm.) (*B*) Plots of fluorescence lifetime of CRhOMe vs. time in active hiPSC-CMs, quantified from the fields of view in *A*. (*C*) Plots of fluorescence intensity of CRhOMe vs. time in active hiPSC-CMs from the same regions as in *A* and *B*. (*Top*) arrow depicts photobleaching; (*Middle*) asterisks depict apparent AP morphological artifact; (*Bottom*) arrowheads show AP artifact. None of these artifacts appears in the lifetime recording. (*D*) Representative recordings of CRhOMe absolute V_mem_ imaging of iCMs treated with 1 µM isoproterenol, which accelerates beat rate. (*E*) Amplitude of iCM APs, as measured by the change in τ of CRhOMe. Data represent 38 recordings from 8 iCM wells. Bin size for histogram was determined by the Freedman-Diaconis rule.

To demonstrate the improved temporal resolution and stability to motion of our absolute V_mem_ sensing platform, we monitored the τ_fl_ of spontaneously beating human-induced pluripotent stem cell–derived cardiomyocytes (hiPSC-CMs). Upon loading CRhOMe or TMCRhZero into iCM monolayers, we observed membrane-localized fluorescence (Fig. 5 and *SI Appendix*, Fig. S12). Because we are using a confocal pinhole larger than 1 airy unit to maximize photon count, some regions of the membrane appear as flat sheets where they traverse the optical section at a near-horizontal angle.

The AP in iCMs is typically on the order of 500 ms in duration. Intensity-based V_mem_ imaging techniques in cardiomyocytes generally use sampling rates between 50 and 500 Hz (44–46). but fluorescence lifetime imaging studies in other systems rarely exceed frame rates of 0.1 Hz. Using TCSPC FLIM, we acquired a raw lifetime data stream at 40 Hz (25 ms acquisition per frame). We binned successive pairs of frames to produce a final recording with a 20 Hz frame rate (Fig. 5*D* and *SI Appendix*, Fig. S13). Because of CRhOMe’s high brightness and photostability, the limiting factor in the achievable frame rate was the speed at which the TCSPC electronics could process photons (the pile-up limit). We did not observe phototoxicity to the sample or drift in the lifetime; this is consistent with our ability to acquire continuously for 15 min in A431 cells (Fig. 4).

We observed a stable lifetime baseline and consistent AP morphology throughout all optical recordings (*SI Appendix*, Fig. S13). We used TMCRhZero to further verify that lifetime is insensitive to motion artifacts from the iCM contraction. Recordings with the voltage-insensitive TMCRhZero derivative are stable throughout the acquisition, despite normal contraction of the iPSCs (*SI Appendix*, Fig. S14).

In contrast to the stability of the lifetime recording, the photon count (fluorescence intensity) from cardiomyocytes shows both a variable baseline and a variable AP waveform (Fig. 5*C* and *SI Appendix*, Fig. S13*B*), presumably from motion artifacts as the cardiomyocytes contract. Even though all wells and fields of view were loaded with the same concentration of dye (500 nM), the baseline intensity varied widely between recordings (*SI Appendix*, Fig. S13*B*). Differences in the amount of membrane in the field of view (Fig. 5*A*), as well as differences in focal plane, are likely responsible for this. Normalizing the photon count into relative fluorescence units (Fig. 5*C* and *SI Appendix*, Fig. S13*C*), as is common with fluorescence intensity imaging, did not fully resolve these artifacts in either baseline drift or AP morphology.

Using CRhOMe fluorescence lifetime as a proxy for absolute V_mem_, we cataloged the AP amplitude, resting τ, and peak τ across 40 iCM recordings (Fig. 5*E* and *SI Appendix*, Figs. S13*D* and S15). We observe a consistent lifetime change of 0.394 ± 0.004 ns (mean ± SEM of 40 recordings across two differentiations). Resting τ (2.37 ± 0.01 ns) and peak τ (2.76 ± 0.01 ns) were somewhat more variable than the AP amplitude for these cells. Because of difficulties associated with electrophysiology in beating sheets of cardiomyocytes, we first attempted to apply the calibration from HEK293T cells to convert these lifetime changes to V_m_. Unfortunately, the sensitivity measured in HEK293T cells (3.1 ps/mV) translates to a non-physical AP amplitude of around 127 mV in cardiomyocytes, based on an average lifetime change of 0.394 ns for each AP. iCM APs are approximately 100 mV in amplitude, although they may range from 80 to 113 mV depending on differentiation conditions (47–50). The discrepancy between this value and the reported iCM AP amplitudes could be due to the previously observed slight dependence of VoltageFluor lifetime sensitivity on cell type (21). We attempted a gramicidin-based calibration in cardiomyocytes, but we found that the ionophore was toxic to cells before V_mem_ became fully depolarized. A future electrophysiological calibration to relate CRhOMe lifetime to a known voltage in iCMs will enable a more quantitative analysis.

To push the temporal resolution of CRhOMe FLIM further, we treated iCMs with the β adrenoreceptor agonist isoproterenol, which increases beat rate (Fig. 5*D*) (51) Under these conditions, we observed spontaneous APs at approximately twice the frequency (~1.2 Hz vs. ~0.6 Hz). Even with this more rapid activity, we were able to record the full AP waveform and a consistent baseline τ. As expected with an increase in beat rate, AP duration shortened in isoproterenol-treated cells. AP amplitude appeared slightly smaller than in treated cells, and baseline τ remained unchanged (Fig. 5*D*). The robustness of the CRhOMe FLIM signal during this treatment suggests that this could be a useful approach for interrogating cardiomyocyte voltage signaling under perturbation or throughout differentiation.

## Outlook/Conclusion

We developed a VF dye, CRhOMe, which provides red to far-red excitation and emission and improved SNR compared to our previous best-in-class BeRST 1 indicator, owing in part to its blue-shifted excitation and emission, which is a better match for excitation sources and peak camera sensitivity. CRhOMe also possesses a monoexponential fluorescence lifetime decay, which substantially simplifies its use in FLIM, requiring fewer photons for an accurate fit (36, 37). This last point will benefit the use of FLIM in photon-starved applications, for example, high-speed FLIM imaging of voltage or imaging from cellular structures smaller than a cell body.

Used in fluorescence intensity mode, CRhOMe enables imaging of AP dynamics in single trials in hippocampal neurons with improved SNR compared to BeRST 1. Used in lifetime mode, CRhOMe reports on physiological plasma membrane hyperpolarization induced by EGF-mediated signaling in mammalian cells with 15 min of continuous illumination, a >fivefold improvement over the first-generation VF-FLIM. CRhOMe is well tolerated in sensitive samples, and coupled with its brightness, fast response time, and monoexponential fluorescence lifetime decay, this allows tracking of cardiac AP dynamics in hiPSC-CMs, in both intensity and lifetime mode. Lifetime imaging of AP dynamics eliminates artifacts associated with moving cells and opens the door to future optical determination of resting membrane potential values in excitable cells. In our current implementation, the FLIM hardware, not the fluorophore, limits the maximum acquisition speed; combining CRhOMe with emerging methods for fast FLIM microscopy (52) would enable even greater acquisition speeds. Overall, CRhOMe is a promising VF dye for the resolution and quantification of membrane potentials in both excitable and non-excitable cells.

## Materials and Methods

Detailed experimental procedures are described in *SI Appendix*. Experimental details for *Fluorescence Microscopy*, *FLIM Acquisition*, *Image Analysis*, *Fitting*, *Spectroscopic Characterization*, *Synthetic Procedures*, and *Supporting Characterization Data* are all included in the *SI Appendix*. All compounds and dyes were synthesized and purified using standard synthetic organic chemistry manipulations. These manipulations are described in detail in *SI Appendix*. Key points are summarized, below.

### Chemical Synthesis and Characterization.

Chemical reagents and solvents (anhydrous) were purchased from commercial suppliers and used without further purification. All reactions were carried out in flame-dried flasks sealed with septa and conducted under a nitrogen atmosphere. Thin layer chromatography (TLC) (silica gel, F254, 250 mm) was performed on precoated TLC glass plates and was visualized by fluorescence quenching under UV light. Flash column chromatography was performed on Silicycle Silica Flash F60 (230 to 400 Mesh) using a forced flow of air at 0.5 to 1.0 bar. NMR spectra were recorded on a Bruker AVB-400 MHz, or at the QB3 Central California 900 MHz NMR Facility. Chemical shifts (δ) are expressed in parts per million (ppm) and are referenced to CDCl_3_ (7.26 ppm, 77.0 ppm) or CD_3_OD (3.31 ppm, 49.0 ppm). Coupling constants are reported as Hertz (Hz). Splitting patterns are indicated as follows: s, singlet; d, doublet; t, triplet; q, quartet; dd, doublet of doublet; m, multiplet.

High-resolution mass spectra (ESI, EI) were measured by the QB3/Chemistry mass spectrometry service at University of California, Berkeley. High-performance liquid chromatography (HPLC) and low-resolution ESI Mass Spectrometry were performed on an Agilent Infinity 1200 analytical instrument coupled to an Advion CMS-L ESI mass spectrometer. The column used for the analytical HPLC was Phenomenex Luna 5 μm C18(2) (4.6 mm I.D. × 150 mm) with a flow rate of 1.0 mL/min. The mobile phases were MQ-H_2_O with 0.05% trifluoroacetic acid (eluent A) and HPLC grade MeCN with 0.05% trifluoroacetic acid (eluent B). Absorbance signals were monitored at 254, 380, 450, 615, and 650 nm.

#### Materials.

Dyes were stored either as solids at room temperature or as 1 mM stock solutions in dimethylsulfoxide (DMSO) at −20 °C. All stock concentrations were determined via the absorbance of the carborhodamine chromophore using a 2501 Spectrophotometer (Shimadzu).

EGF (Peprotech) was made up as a 1 mg/mL stock solution in water and stored at −80 °C. Gramicidin (Sigma-Aldrich) was purchased as a mixture of A, B, C, and D from *Bacillus brevis*. Then, 1 µg/mL stocks of gramicidin were made up in DMSO and stored at −20 °C. Isoproterenol was a gift from the Healy lab at UC Berkeley and was stored as a 10 mM stock in DMSO at −20 °C.

### Cell Culture.

#### Immortalized cell line culture.

HEK293T and A431 cells were obtained from the UC Berkeley Cell Culture Facility. Both cell lines were verified by short tandem repeat profiling and were tested routinely for mycoplasma. Cell lines were discarded after 25 passages. Cells were maintained in complete Dulbecco’s modified Eagle’s medium (DMEM, Gibco, Thermo Fisher Scientific) supplemented with 4.5 g/L glucose, 2 mM GlutaMAX (Gibco), and 10% fetal bovine serum (FBS, Seradigm) in a 37 °C humidified incubator at 5% CO_2_. Cells were passaged into fresh complete media every few days following dissociation with trypsin-EDTA (Gibco, 0.05% for HEK293T, 0.25% for A431). Residual trypsin was removed from A431 cells by centrifugation for 5 min at 300 × g.

For imaging experiments, cells were plated onto prepared poly-D-lysine (PDL)-coated coverslips. Coverslips (#1.5, either 12 mm or 25 mm diameter, Electron Microscopy Sciences) were acid washed for 2 to 5 h in 1 M HCl. Coverslips were then washed three times overnight in 100% ethanol, followed by three times overnight in MilliQ (Millipore) purified water. Coverslips were sterilized by heating for 2 to 3 h in a glassware oven to 150 °C. Prior to seeding of cells, coverslips were incubated in 1× PDL (Sigma-Aldrich, made as a 0.1 mg/mL solution in phosphate-buffered saline with 10 mM Na_3_BO_4_) for 1 to 10 h and then washed twice with sterile water and twice with 1× Dulbecco’s PBS (Gibco).

For probe loading and gramicidin treatment experiments, HEK293T were seeded onto prepared coverslips in complete DMEM at a density of 42 to 52 × 10^3^ cells per cm^2^ (in a 6-well or 24-well tissue culture plate, Corning) and imaged approximately 24 h after plating. For electrophysiology experiments, HEK293T were seeded at 26,000 cells/cm^2^ in low glucose DMEM (Gibco; 1 g/L glucose, 1 mM sodium pyruvate, 2 mM GlutaMAX, 10% FBS) and used 12 to 24 h after plating.

A431 cells were serum-deprived prior to use. Two days before imaging experiments, cells were trypsinized and suspended in complete media with 10% FBS. The cells were then spun down for 5 min at 500 × g and resuspended in low serum DMEM (4.5 g/L glucose, 2 mM GlutaMAX, 2% FBS). Cells were then seeded onto PDL-coated glass coverslips at a density of 83,000 cells/cm^2^ in the low serum DMEM. In addition, 3.5 to 5.5 h prior to imaging, medium was exchanged for serum-free DMEM (4.5 g/L glucose, 2 mM GlutaMAX). Cells were loaded with dye after 4 to 5.5 h in the serum-free media.

## Supplementary Material

Appendix 01 (PDF)

## Data Availability

All study data are included in the article and/or *SI Appendix*.
